# Development of quantitative PCR for the detection of *Alkalilimnicola ehrlichii*, *Thioalkalivibrio sulfidiphilus* and *Thioalkalibacter halophilus* in gas biodesulfurization processes

**DOI:** 10.1186/s13568-019-0826-1

**Published:** 2019-07-05

**Authors:** Karine Kiragosyan, Pieter van Veelen, Suyash Gupta, Agnieszka Tomaszewska-Porada, Pawel Roman, Peer H. A. Timmers

**Affiliations:** 1 Wetsus, European Centre of Excellence for Sustainable Water Technology, Oostergoweg 9, 8911 MA Leeuwarden, The Netherlands; 2 Environmental Technology, Wageningen University, P.O. Box 17, 6700 AA Wageningen, The Netherlands; 3 Microbial Systems Ecology, Department of Freshwater and Marine Ecology, Institute for Biodiversity and Ecosystem Dynamics, University Amsterdam, P.O. Box 94240, 1090 GE Amsterdam, The Netherlands; 4 Laboratory of Microbiology, Wageningen University, P.O. Box 8033, 6700 EH Wageningen, The Netherlands

**Keywords:** Sulfur-oxidizing bacteria, qPCR, Primers, Gas biodesulfurization

## Abstract

Chemolithoautotrophic sulfur-oxidizing bacteria (SOB) are crucial key players in biotechnological processes to remove hydrogen sulfide from sour gas streams. Several different haloalkaliphilic SOB have been detected and isolated from lab- and full-scale facilities, which all performed differently considering end product yields (sulfur and sulfate) and conversion rates. Understanding and regulating bacterial community dynamics in biodesulfurization processes will enable optimization of the process operation. We developed quantitative PCR (qPCR) assays to quantify haloalkaliphilic sulfur-oxidizing gammaproteobacterial species *Alkalilimnicola ehrlichii*, *Thioalkalivibrio sulfidiphilus*, and *Thioalkalibacter halophilus* that dominate bacterial communities of biodesulfurization lab- and full-scale installations at haloalkaline conditions. The specificity and PCR efficiency of novel primer sets were evaluated using pure cultures of these target species. We further validated the qPCR assays by quantification of target organisms in five globally distributed full-scale biodesulfurization installations. The qPCR assays perform a sensitive and accurate quantification of *Alkalilimnicola ehrlichii*, *Thioalkalivibrio sulfidiphilus* and *Thioalkalibacter halophilus*, thus providing rapid and valuable insights into process performance and SOB growth dynamics in gas biodesulfurization systems.

**Electronic supplementary material:**

The online version of this article (10.1186/s13568-019-0826-1) contains supplementary material, which is available to authorized users.

## Introduction

Sulfur-oxidizing bacteria (SOB) are microorganisms that naturally occur in highly saline and alkaline environments such as soda lakes (Sorokin and Kuenen 2005; Sorokin et al. 2013). SOB are known to be present in complex and species-rich consortia of microorganisms involved in both anaerobic and aerobic processes (Janssen et al. 2009) and can grow chemolithoautotrophically using inorganic sulfur compounds as electron donor and CO_2_ as carbon source (Ghosh and Dam 2009). These chemolithoautotrophic SOB are specifically enriched in biotechnological processes to remove hydrogen sulfide (H_2_S) from industrial gas streams by producing sulfur (Van Den Bosch et al. 2007; Sorokin et al. 2011). Biodesulfurization processes of the Thiopaq^®^ family are operated under haloalkaline conditions, i.e. high pH (≥ 8.5) and high soda concentrations (1 M Na^+^) (Van Den Bosch et al. 2007).

Since 2009, the number of gas biodesulfurization installations increased globally (Driessen et al. 2011). All full-scale installations operate at different process conditions i.e. pH, salinity, oxidation–reduction potential (ORP) and treat feed gas of a various compositions (i.e. presence of different organic carbon compounds or contaminants such as thiols and BTEX) (Kiragosyan et al. 2019). To investigate which factors ensure stable process operation, a number of lab- and full-scale gas biodesulfurization installations have been monitored on the microbial community composition and process conditions. In sulfide-fed lab-scale installations, *Thioalkalivibrio sulfidiphilus* was the dominant SOB (Sorokin et al. 2008; Kiragosyan et al. 2019). In other lab-scale installations with sulfide feed gas supplemented with thiols, *Alkalilimnicola* sp. and *Thioalkalibacter* sp. were found to dominate (Roman et al. 2016). The addition of these toxic thiols not only affected SOB community composition, but alterations of the biodesulfurization process conditions can also cause a community change. For example, changes in the bioreactor design or addition of an extra bioreactor can also result in SOB community composition shift (De Rink et al. 2019).

In most cases, these SOB community dynamics were monitored using 16S rRNA gene amplicon sequencing. This method provides only estimates of relative taxon abundances and it is time consuming and costly. Monitoring SOB dynamics with quantitative PCR (qPCR), would provide absolute numbers of key SOB species in biodesulfurization processes, and is relatively fast, less costly and results can be analyzed and interpreted with ease. The ability to monitor SOB species dynamics rapidly and inexpensively will help to monitor the key population dynamics and to optimize biodesulfurization process efficiency. qPCR is widely used for microbial quantification in many types of environmental studies (Filion 2012), but so far no SOB-specific primers are currently available to monitor dynamics of relevant SOB species. The qPCR specificity for different SOB species depends on several parameters: the primer sequences for the target, time and temperature of primer annealing, annealing, amplicon product size, concentration of Mg^2+^, dNTPs, fidelity of enzymes, and the purity of the DNA sample (Robertson and Walsh-Weller 1998).

In this work we designed target-specific primers and developed specific qPCR assays to monitor absolute abundances of the three most dominant haloalkaliphilic SOB species found in operational biodesulfurization Thiopaq^®^ installations until now: *Alkalilimnicola ehrlichii*, *Thioalkalivibrio sulfidiphilus* and *Thioalkalibacter halophilus*. The resulting quantitative measures provide insights in species growth dynamics and interactions. Hence, developed quantitative PCR assays can be used to establish relationships between the operational conditions and the biological community in biodesulfurization processes in order to establish stable SOB communities that ensure predictable and stable process conditions.

## Materials and methods

### Microbial sludge sampling and sample preparation

Biomass from a lab-scale, fed-batch biodesulfurization system, which was fed with H_2_S and methanethiol gas, was obtained after 76 days of continuous operation. H_2_S gas was continuously supplied at a loading rate of 58.15 mM S day^−1^, whereas methanethiol loading rate was stepwise (add steps from 0 to 2 mM) increased for biomass acclimatization during the 76 days to a maximum concentration of 2 mM S day^−1^. On the last day of operation, microbial sludge was sampled and centrifuged for 15 min at 16,000*g* to obtain the cell pellet. The cell pellet was washed twice with 0.5 M Na^+^ buffer solution (pH 8.5) to prevent cell lysis and was subsequently stored at − 80 °C until DNA extraction.

### DNA isolation and purification

Genomic DNA was extracted using the DNeasy PowerLyzer PowerSoil Kit (Qiagen, Venlo, the Netherlands) following the manufacturer’s instructions. After DNA extraction, DNA was purified with the DNA Clean and Concentrator kit (Zymo Research, Irvine, CA, USA). Extracted DNA was quantified using the QuantiFluor dsDNA system on a Quantus™ fluorometer (Promega, Leiden, the Netherlands). DNA quality was evaluated using gel electrophoresis.

### Clone library construction and sequencing

To design primers targeting the most dominant SOB species present in the established mixed SOB population, full-length 16S rRNA gene sequences recovered from the community DNA. The full 16S rRNA gene was amplified from the extracted and purified DNA using universal bacterial primers 27F (5′-GTTTGATCCTGGCTCAG-3′) (Felske and Weller 2004) and 1492R (5′-CGGCTACCTTGTTACGAC-3′) (Lane 1991). The PCR program started with initial denaturation (95 °C for 2 min) followed by 30 cycles of denaturation for 30 s at 95 °C, annealing for 40 s at 52 °C, elongation for 1.30 min at 72 °C, and with a final 7 min elongation at 72 °C. The PCR products were again purified with the DNA Clean and Concentrator kit and ligated into the pGEM-T Easy Vector System, according to the manufacturer’s instructions (Promega, City, the Netherlands). The ligation mixture was used to transform *Escherichia coli* JM109 competent cells (Promega, City, the Netherlands). Colonies were picked and DNA was extracted and sent for Sanger sequencing to BaseClear B.V. (Leiden, the Netherlands). Obtained forward and reverse sequences were assembled into contigs, and ends were quality trimmed with the DNA Baser software (v4, Heracle Biosoft, www.DnaBaser.com, Romania). Subsequently, sequences were cleaned by cutting off primer sequences and were screened for vector contamination using VecScreen (NCBI, MD, USA).

### Target species-specific primer design

**Fig. 1:**
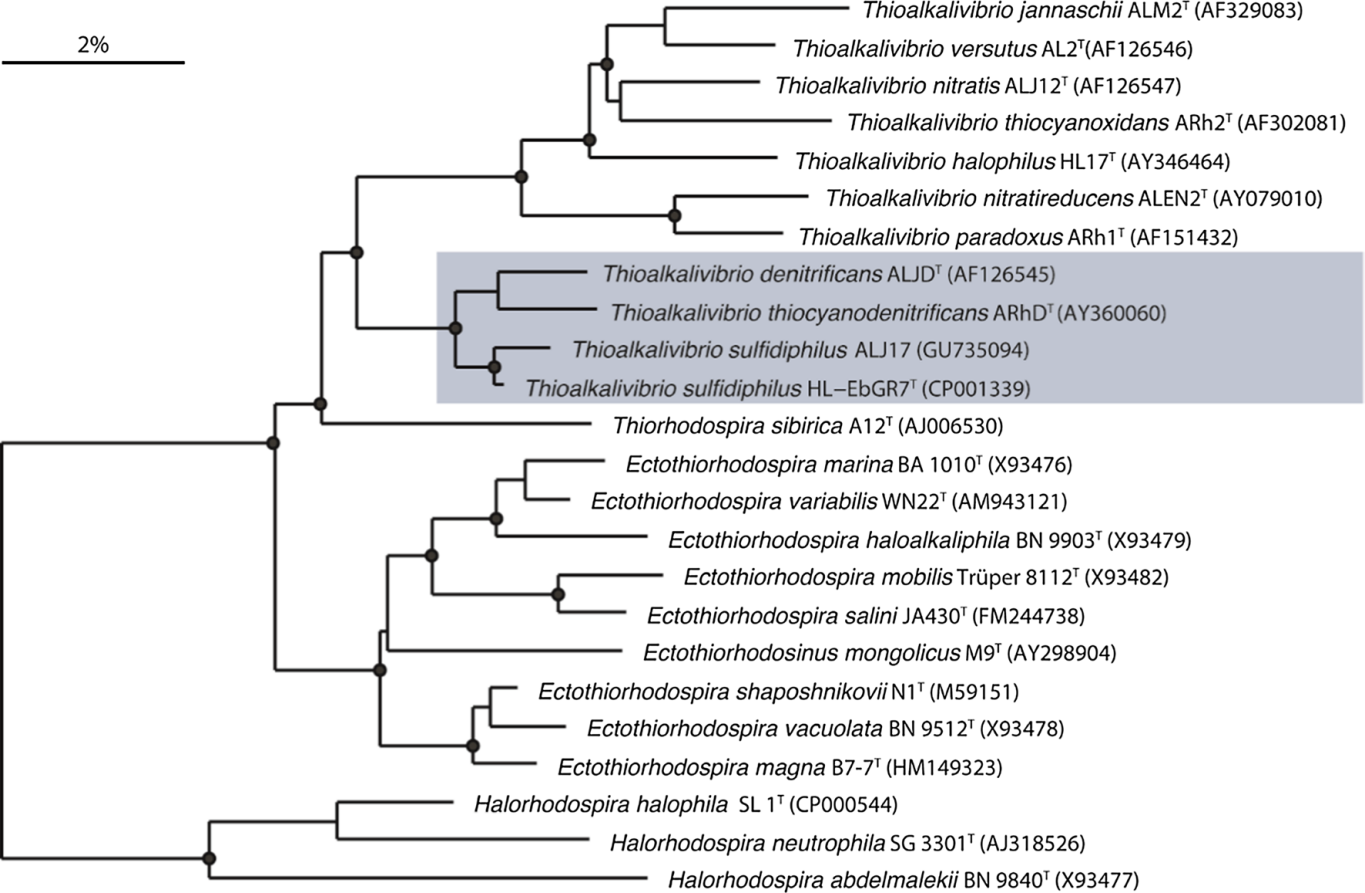
Phylogenetic position of *Thioalkalivibrio sulfidiphilus* in the cluster for which primers for *Thioalkalivibrio* spp. were designed (adapted from Sorokin et al. 2012). Bar indicates 2% sequence divergence

The full length 16S rRNA gene sequences were aligned and taxonomically classified using the SINA alignment tool (v1.2.11) according to the global SILVA alignment (Pruesse et al. 2007). Sequence identity was also investigated with the online nucleotide BLAST tool (Madden 2002). Aligned sequences of the species of interest (*Thioalkalivibrio sulfidiphilus*, *Alkalilimnicola ehrlichii* and *Thioalkalibacter halophilus*) were merged with the SILVA 16S rRNA gene database version SSU rl 28 Ref NR (Quast et al. 2013) using the ARB software package (arb-6.0.6) (Ludwig et al. 2004). Primer sets were designed based on the highest specificity (100% with 0 mismatches) for *Alkalilimnicola ehrlichii* and *Thioalkalibacter halophilus* species. For *Thioalkalivibrio* spp., primers were designed to target the *Thioalkalivibrio denitrificans* cluster (highlighted in Fig. 1). The designed primers were then validated in silico using the TestProbe and TestPrime services of Silva (Klindworth et al. 2013), Primer-BLAST (Ye et al. 2012) and in silico PCR (http://insilico.ehu.es/user_seqs/). After validation, designed primer sequences were ordered from Biolegio (Nijmegen, the Netherlands).

### In vitro primer evaluation on target species pure cultures

To be able to test the specificity of the designed primers and to optimize the qPCR protocol, every designed primer set was tested using pure cultures. Two strains used in this study were obtained from the German Collection of Microorganisms and Cell Cultures (DSMZ): *Alkalilimnicola ehrlichii* strain MLHE-1 (DSM-17681) and *Thioalkalibacter halophilus* strain ALCO 1 (DSM-19224). *Thioalkalivibrio sulfidiphilus* strain HL-EbGr7 was provided from the personal collection of Prof. Dr. Gerard Muyzer (Amsterdam University, the Netherlands).

### qPCR assay optimization

For the optimization of qPCR assays the workflow was as follows:

1. Firstly, the annealing temperature was optimized based on the theoretical melting temperature (T_m_) via gradient PCR (± 10 °C). In this step, pure cultures were used as positive controls for the selected primer sets. After each temperature gradient run, gel electrophoresis (GE) was performed to confirm the size of the PCR product(s) based on expected insert lengths of the developed primer sets.
2. For each primer set, primer specificity for a target species was assessed by simultaneous testing against the non-target cultured pure strains (*Thioalkalivibrio sulfidiphilus* strain HL-EbGr7, *Thioalkalivibrio denitrificans* strain ALJD (DSM-13742), *Alkalilimnicola ehrlichii* strain MLHE-1 and *Thioalkalibacter halophilus* strain ALCO 1) and cultured clones found in our cloned biomass [*Thioalkalimicrobium* spp., *Halomonas* sp. HB. Br (GU228481)] at the selected optimal annealing temperature for each primer set. When bright bands of the positive target strain were visible on GE and no bands of non-target strain negative controls appeared, fine-tuning of the optimal annealing temperature was continued with a narrower temperature gradient ranging between ± 3 °C.
3. To further assess primer specificity, melting curve analysis was performed (50–95 °C, with 0.5 °C increments) using quantitative PCR (Bio Rad CFX96 Touch™, City, the Netherlands) with SYBR^®^ Green fluorescent dye (Bio Rad, the Netherlands). The qPCR reaction volume was 20 µl with 0.33 pmol µl^−1^ of forward and reverse primers. Melt curves revealed target-specific product formation at the determined optimal annealing temperatures for all three primer sets (see Additional file 1: Figures S1, S2 and S3). The optimal annealing temperatures for cultured positive control strains were then applied in the qPCR analysis of bioreactor experimental samples. For primer set *Thioalkalivibrio* spp. Thio-6F and Thio-8R, the optimal T_m_ was 55 °C, for Alkali-4AF and Alkali-6BR T_m_ was 53 °C, and for Tab-137-G_F and Tab-210R best T_m_ was at 66 °C.
4. To verify that our developed primer sets specifically amplified the target species in our experimental samples, melting curves of experimental samples were analyzed for qPCR amplicon specificity, and then a random subset of (n = 4 to 6 per primer set) qPCR products were sent for Sanger sequencing to BaseClear B.V. (Leiden, The Netherlands). Sequences were assembled using the DNA Baser software (v4, Heracle Biosoft, www.DnaBaser.com, Romania) and identified with BLAST (Madden 2002). When qPCR amplicons from the experimental samples were positively identified as the target species, we continued with the preparation of standard curves using the DNA of the pure cultures.
5. As a positive control, cultured pure strains were used in target species-specific qPCR assays to establish a standard curve in order to quantify each of their copy numbers in the experimental samples. The concentrations of the positive control DNA were measured using QuantiFluor dsDNA systems and a Quantus™ fluorometer (Promega, the Netherlands). Positive controls were then serially diluted in tenfold dilutions ranging between 10^6^ and 10^1^ copies μl^−1^ of the 16S rRNA gene. These serial dilutions were used to generate standard curves which allowed minimal reaction efficiencies of 90–100% and 0.997 < R^2^ < 0.999.

### Validation of the developed qPCR assay

To validate the developed qPCR methods, we used biomass from different five full-scale gas biodesulfurization installations (Table 1). Additional research focusing on the microbial community compositions of these full-scale installations was based on 16S rRNA gene amplicon sequencing (Kiragosyan et al. 2019). The applicability of the qPCR assay was validated by comparing qPCR-based relative target abundances with relative abundances obtained from the 16S rRNA gene amplicon sequence data. To calculate relative target abundances, total bacterial 16S rRNA gene copy abundance was quantified using the universal bacterial primer set 338F/518R (Lane 1991; Muyzer et al. 1993) and an in-house protocol (Pallares-Vega et al. 2019).

**Table 1:** A brief description of the full-scale biodesulfurization installations, and averaged operational parameters

Location	Industry	Sour gas composition	Sample ID
Eerbeek (NL)	Paper mill	Biogas, 0.7% H_2_S	Paper mill—1
Zülpich (DE)	Paper mill	Biogas, 0.5% H_2_S	Paper mill—2
Amersfoort (NL)	Landfill waste	Landfill gas, 0.3% H_2_S	Landfill
Southern Illinois (USA)	Oil and gas	Associated gas, 1–5% H_2_S, 50–200 ppm VOSC^a^	Oilfield—1
Sulawesi (ID)	Oil and gas	Acid gas 80–90%, 10–20% H_2_S, X ppm thiols	Oilfield—2

^a^VOSC—volatile organic sulfur compounds, e.g., thiols and diorganopolysulfides

### Accession number

The EMBL-EBI accession numbers of the full-length 16S rRNA clonal gene sequences of *Alkalilimnicola ehrlichii*, *Thioalkalivibrio sulfidiphilus* and *Thioalkalibacter halophilus* are LR214448–LR214450 in the project number PRJEB30777. 16S rRNA gene amplicon sequences from the full-scale installations are deposited under EMBL-EBI project accession number PRJEB27163 and PRJEB32000.

## Results

### Primers evaluation and qPCR assay optimization

For each of the three targets, we designed three primer sets for which we subsequently optimized the qPCR protocols. The properties of the primers that were tested in silico and in vitro are summarized in Table 2.

**Table 2:** Description of designed species-specific primers description

Primer ID	Primer sequence	*E. coli* position	Theoretical T_m_ (°C)	Length (bp)	GC content (%)
Thio-6F	AGG GCT AGA GTT TGG TAG	647	52	18	50
Thio-8R	AGA GGC ATA ATC CTC CCA	834	54	18	50
Alkali-4AF	GTT AAT AGC CGT GGG TCT	462	54	18	50
Alkali-6BR	TAC CAG ACT CTA GCC CGA	646	56	18	56
Tab-137-G_F	CTT AGG TGG GGG ATA ACA CG	137	57	20	55
Tab-210R	ATC CTT TGG CGC GAG GTC CG	210	65	20	65

F, forward; R, reverse; Thio, *Thioalkalivibrio* spp.; Alkali, *Alkalilimnicola ehrlichii*; Tab, *Thioalkalibacter halophilus*

In silico amplification demonstrated that primer set Thio-6F/Thio8-R quantified *Tv. sulfidiphilus* and closely-related *Tv. denitrificans.* Empirical qPCR experiments and subsequent melt-curve analysis on DNA extracted from pure cultures of *Tv. sulfidiphilus* and *Tv. denitrificans* correspondingly showed amplification of both species at the same annealing temperature (Additional file 1: Figure S1). qPCR assays for *Alk. ehrlichii* and *Th. halophilus* showed species-specificity in silico and in vitro (Additional file 1: Figures S2 and S3). Yet, our temperature gradient experiment conducted for the *Alk. ehrlichii*-targeting primer set Alkali-4AF/Alkali-6BR suggested amplification of non-target DNA at temperatures exceeding 55 °C. Melt curve analysis of this temperature gradient demonstrated that non-target amplification reduced with decreasing temperatures. A subsequent temperature gradient including lower temperatures revealed that at 53 °C, the desired specificity for *Alkalilimnicola ehrlichii* was reached. Optimal reaction parameters for the developed qPCR assays were initial denaturation for 5 min at 95 °C followed by 30 amplification cycles of denaturation for 10 s at 95 °C, annealing for 30 s at 53 °C for *Alkalilimnicola ehrlichii*, 55 °C for *Thioalkalivibrio sulfidiphilus* and 66 °C for *Thioalkalibacter halophilus*.

### Validation of the developed qPCR assay

**Fig. 2:**
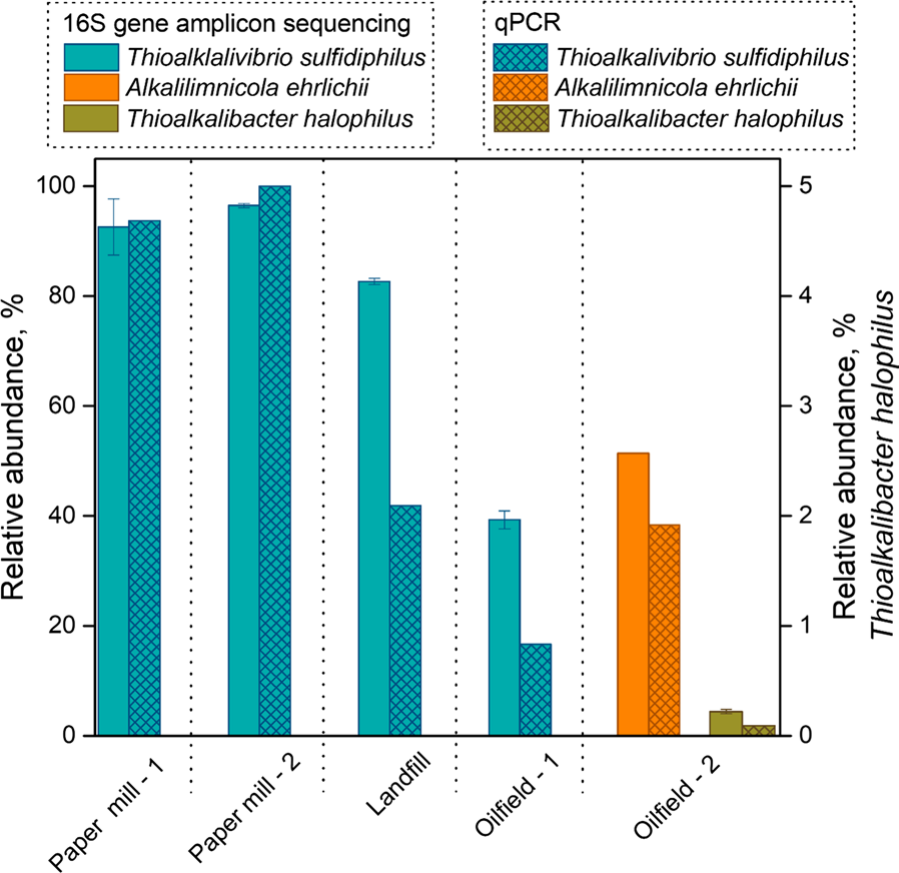
Comparison of relative taxon abundances using 16S rRNA gene amplicon sequencing (NGS) and species-specific qPCR from five full-scale gas biodesulfurization installations. The results represent the average value per full-scale biodesulfurization installation for each of the target species that was positively detected. Error bars represents the standard deviation of technical duplicates. Amplicon sequence data are modified from Kiragosyan et al. 2019

To validate the applicability of the developed qPCR assays, we quantified absolute abundances of the target species and total bacteria in five full-scale gas biodesulfurization installations geographically distributed across Europe, Asia, and North America (Table 1). Our results demonstrated that the qPCR-based relative species abundances (i.e. ratio species-specific to total bacterial 16S rRNA amplicon count) are analogous to the relative abundances obtained by16S rRNA gene amplicon sequencing (i.e. ratio taxon-specific to total read count) of the microbial community structure in the full-scale biodesulfurization installations (Fig. 2). In both Paper mill installations, the relative abundance of *Thioalkalivibrio* spp. quantified by qPCR and amplicon sequence data are of the same magnitude. However, in three other full-scale installations quantified abundances by qPCR assays differed from amplicon sequencing data. Relative abundances of *Thioalkalivibrio* spp. detected by qPCR in Oilfield and Landfill plants were two times less than the relative abundance of *Tv. sulphidophilus* in amplicon sequencing data (Fig. 2). The same twofold difference was found for less abundant *Th. halophilus* in Oilfield—2 installation (0.22% vs. 0.09%) (Fig. 2). Collectively, these results indicate that the developed qPCR assays can be applied for detection and quantification of *Alkalilimnicola ehrlichii*, *Thioalkalivibrio sulfidiphilus* and *Thioalkalibacter halophilus* in various gas biodesulfurization installations.

## Discussion

Developed qPCR assays for fast and accurate quantification of *Alkalilimnicola ehrlichii*, *Thioalkalivibrio sulfidiphilus* and *Thioalkalibacter halophilus* were optimized to achieve maximal specificity and sensitivity to detect potentially low abundance of target species. For *Alk. ehrlichii* and *Th. halophilus*, the designed primers achieved 100% specificity by allowing 0 mismatches in the primer binding region. For *Thioalkalivibrio* spp., primers were designed for a subcluster of this genus (Fig. 1). *Thioalkalivibrio sulfidiphilus* HL-EbGR7 is genetically related to another *Tv. sulfidiphilus* ALJ17 and to *Tv. denitrificans* (Sorokin et al. 2012; Ahn et al. 2017). However, our cloning results of the biodesulfurization process sludge and finding of Sorokin et al. (2012) indicate that only *Tv. sulfidiphilus* was found in the lab- and full-scale installations. Hence, we can conclude that the designed primer set quantified *Tv. sulfidiphilus* in the samples as it is the only *Thioalkalivibrio* species detected and dominantly present at low salt conditions in the gas biodesulfurization lab- and full-scale installations. The targeted sub-cluster contains other two closely related species, which are *Tv denitrificans* and *Tv. thiocyanodenitrificans* (Sorokin et al. 2012). The designed primer set Thio6F/Thio8R is specific for *Tv. sulfidiphilus* and *Tv. denitrificans*, whereas *Tv. thiocyanodenitrificans* was not tested (see Additional file 1: Figure S1). However, *Tv. denitrificans* and *Tv. thiocyanodenitrificans* have never been detected as thiodenitrifying conditions are not provided in the full-scale biodesulfurization process. It is therefore confirmed that the primer set Thio6F/Thio8R only quantified *Thioalkalivibrio sulfidiphilus* in the experimental samples from biodesulfurization full- and lab-scale installations.

Optimization of the quantitative real-time PCR protocols enhanced the specificity of the designed primer sets in experimental samples. Conventionally, regular PCR temperature gradients are advisable to be performed with subsequent visualization of product bands using gel electrophoresis. However, the detection by agarose gel electrophoreses is limited for low (expected) product concentrations (Smith and Osborn 2009; Garibyan and Avashia 2013). For the Alkali-4AF/Alkali-6BR primer set targeting *Alkalilimnicola ehrlichii*, we reached specificity at a lower than expected annealing temperature. Similarly, Sipos et al. (2007) found that three universal bacterial primers showed better performance at lower temperatures based on temperature gradients with varying annealing temperatures (47 to 61 °C). Furthermore, Ischii and Fukui (Ishii and Fukui 2001) also found that at low temperatures, mismatch biases of primers were reduced. Apparently, decreasing annealing temperatures can yield improved target specificity of qPCR assays, which proved a beneficial property for the *Alkalilimnicola ehrlichii* assay. In addition, the reverse primer with relatively high GC content Alkali-6BR (56%, Table 2), was more specific for detection of *Alkalilimnicola ehrlichii* at lower temperatures.

Sequencing based methods give the possibility to resolve the community composition of complex experimental samples, where further qPCR assay can be complementary applied to answer more profound questions on population dynamics of a specific target organism. In our work, we showed that with use of both techniques similar results can be achieved, which confirms the accuracy of the developed qPCR assays. Relative abundance estimates for dominant species between qPCR and 16S amplicon sequence data were comparable in both Paper mill installations, while in other full-scale installations there was a two-fold difference. Observed difference might be caused by the use of different universal primer sets for 16S rRNA gene quantification in qPCR and NGS, because no primer set is truly universal (Leray et al. 2013). Primers have different affinity for different taxonomic groups what prevents detection of certain operational taxonomic units (OTUs) in NGS (Leray and Knowlton 2017). This results in biased relative abundances especially within complex samples (Piñol et al. 2015). In addition, variation between qPCR and NGS counts can be explained by a PCR bias (in NGS) introduced by less dominant species (Stokell et al. 2015). Hence, we suggest that it is reasonable to expect that the final number of reads for the target species is higher using universal primers after a defined number of exponential amplification cycles. Detection limits of rare taxa are strongly dependent on the sequencing depths of sample (i.e. number of sequence reads per sample). In 16S rRNA gene amplicon sequencing methods, competition for primers occurs between rare and abundant taxa, where most likely abundant taxa outcompete amplification of rare taxa (Forde and O’Toole 2013). With qPCR however, species-specific absolute quantification of rare and abundant taxa can be done.

The development of three novel qPCR assays allows for accurate, sensitive, fast and cost-efficient quantification of *Alkalilimnicola ehrlichii*, *Thioalkalivibrio* spp. and *Thioalkalibacter halophilus* in complex samples, such as lab- and full-scale gas biodesulfurization installations. The presented qPCR assays will have ample applicability for monitoring dynamics of key SOB species in the gas biodesulfurization process, especially in the presence of common process perturbations such as thiols. Moreover, established dynamics will allow us to expand our understanding of the gas biodesulfurization process and thus, will enable us to improve process performance.

## Additional file

**Additional file 1: Figure S1.** Melting curve analysis of qPCR products of 16S rRNA gene of *Thioalkalivibrio* genus with use of SYBR Green. **Figure S2.** Melting curve analysis of qPCR products of 16S rRNA gene of *Alkalilimnicola ehrlichii* with use of SYBR Green. **Figure S3.** Melting curve analysis of qPCR products of 16S rRNA gene of *Thioalkalibacter halophilus* with use of SYBR Green.

## Data Availability

The data supporting the conclusions of this article are included within the article. Data and materials can also be requested from the corresponding author.
