# Supplementary material for: Development of quantitative PCR for the detection of Alkalilimnicola ehrlichii, Thioalkalivibrio sulfidiphilus and Thioalkalibacter halophilus in gas biodesulfurization processes
Source: AMB Express. 2019 Jul 5;9:99. doi: 10.1186/s13568-019-0826-1 (PMC6611852; doi:10.1186/s13568-019-0826-1)
Supplement: Supplementary file 1 — Additional file 1: Figure S1. Melting curve analysis of qPCR products of 16S rRNA gene of Thioalkalivibrio genus with use of SYBR Green. Figure S2. Melting curve analysis of qPCR products of 16S rRNA gene of Alkalilimnicola ehrlichii with use of SYBR Green. Figure S3. Melting curve analysis of qPCR products of 16S rRNA gene of Thioalkalibacter halophilus with use of SYBR Green. [file 13568_2019_826_MOESM1_ESM.docx]

*AMB Express*

**Development of quantitative PCR for the detection of *Alkalilimnicola ehrlichii*, *Thioalkalivibrio* *sulfidiphilus* and *Thioalkalibacter halophilus* in gas biodesulfurization processes**

Karine Kiragosyan^1,2^, Pieter van Veelen^1^, Suyash Gupta^1,3^, Agnieszka Tomaszewska-Porada^1^, Pawel Roman^1^, Peer H.A. Timmers^1,4^

^1^ Wetsus, European Centre of Excellence for Sustainable Water Technology, Oostergoweg 9, 8911 MA Leeuwarden, the Netherlands

^2^ Environmental Technology, Wageningen University, P.O. Box 17, 6700 AA Wageningen, the Netherlands

^3^ Microbial Systems Ecology, Department of Freshwater and Marine Ecology, Institute for Biodiversity and Ecosystem Dynamics, University Amsterdam, P.O. Box 94240, 1090 GE Amsterdam, the Netherlands

^4^Laboratory of Microbiology, Wageningen University, P.O. Box 8033, 6700 EH Wageningen, The Netherlands

Karine Kiragosyan

+31 (0) [58 284 3000](https://www.google.com/search?q=wetsus&rls=com.microsoft:en-US:%7breferrer:source?%7d&ie=UTF-8&oe=UTF-8&sourceid=ie7&rlz=1I7ADFA_ruRU483)

karine.kiragosyan@wetsus.nl


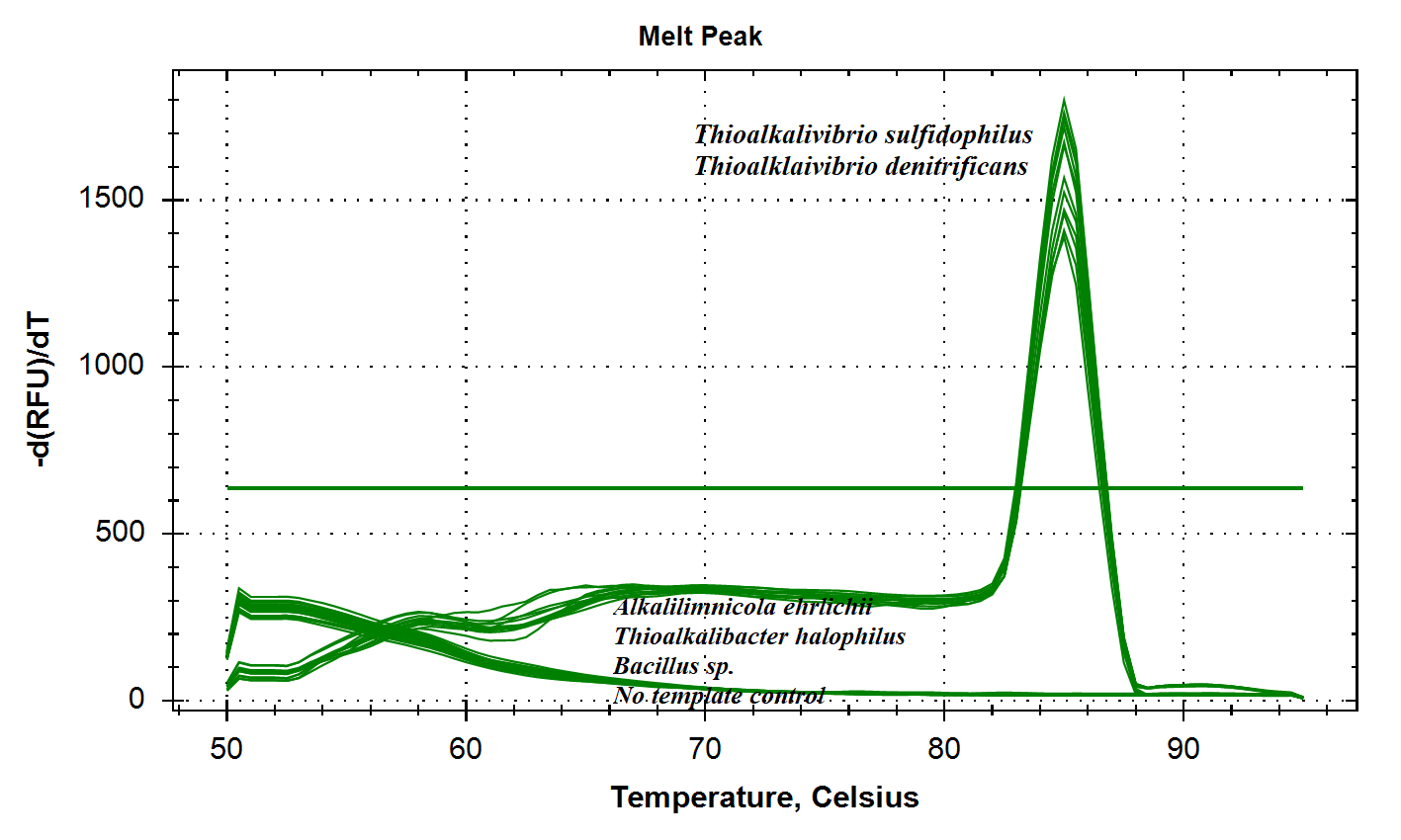


**Figure S1.** Melting curve analysis of qPCR products of 16S rRNA gene of *Thioalkalivibrio* genus with use of SYBR Green


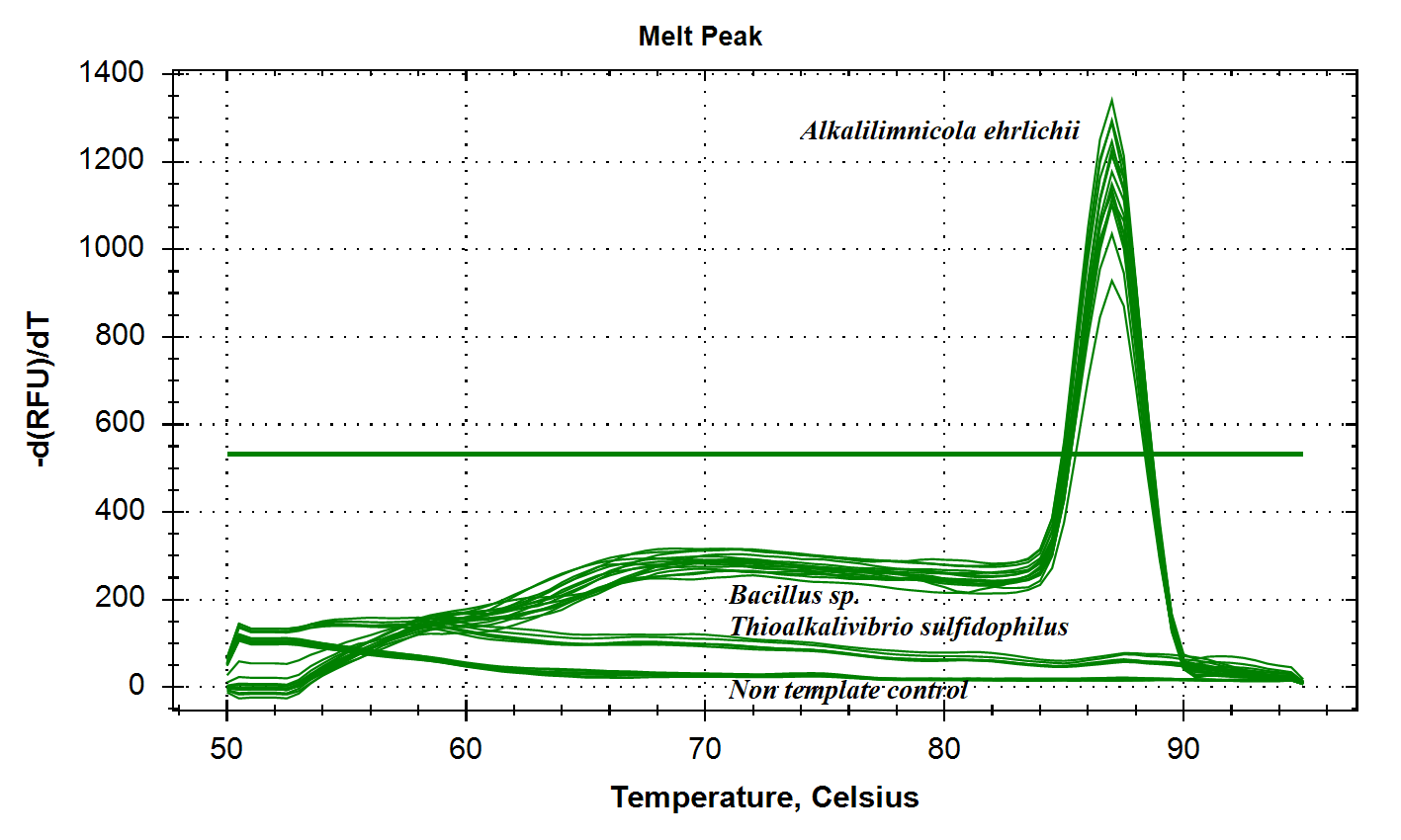


**Figure S2.** Melting curve analysis of qPCR products of 16S rRNA gene of *Alkalilimnicola ehrlichii* with use of SYBR Green


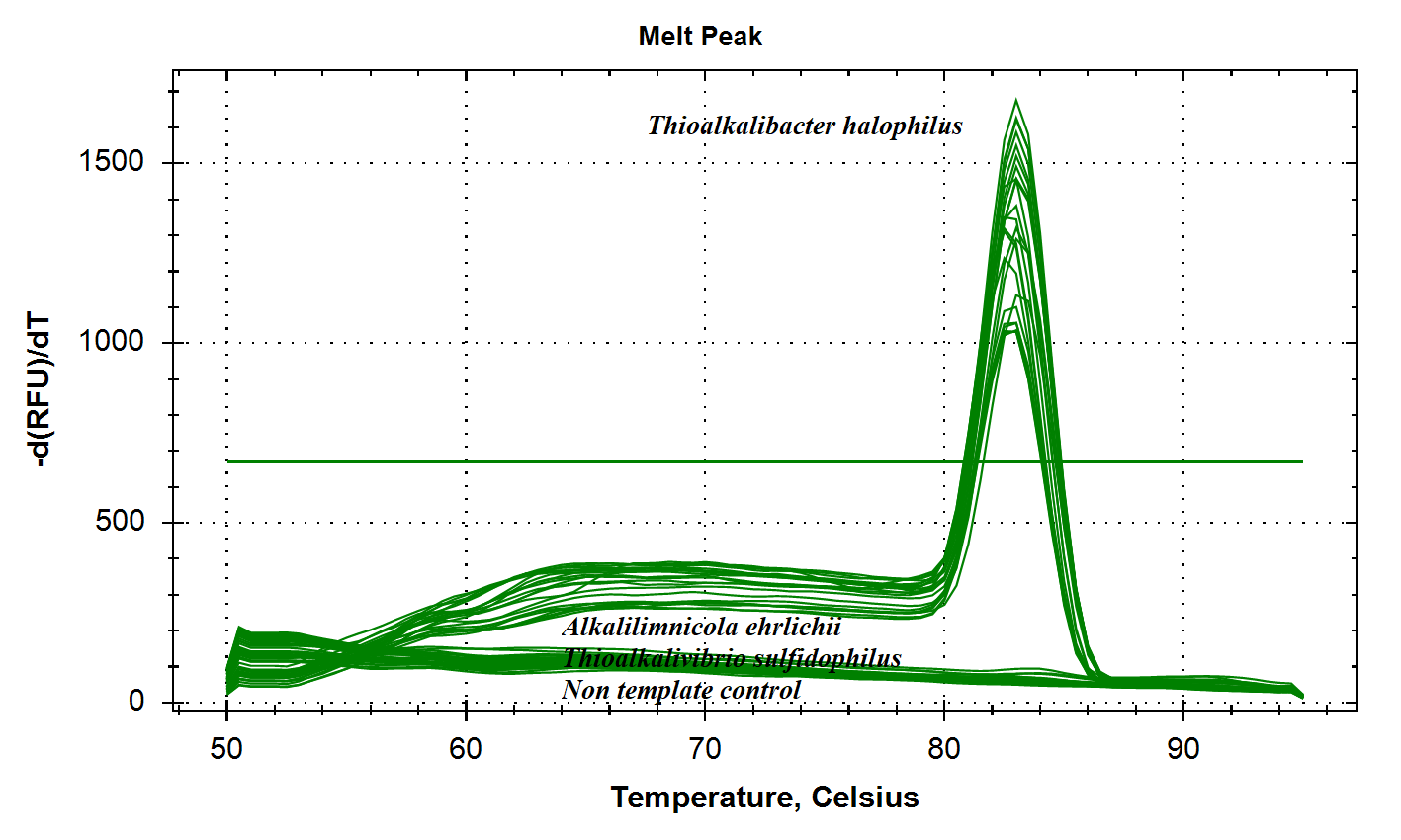


**Figure S3.** Melting curve analysis of qPCR products of 16S rRNA gene of *Thioalkalibacter halophilus* with use of SYBR Green
